# Stereotactic body radiation therapy for the treatment of early-stage minimally invasive adenocarcinoma or adenocarcnioma *in situ* (formerly bronchioloalveolar carcinoma): a patterns of failure analysis

**DOI:** 10.1186/1748-717X-8-4

**Published:** 2013-01-03

**Authors:** Shahed N Badiyan, Andrew J Bierhals, Jeffrey R Olsen, Kimberly M Creach, Adam A Garsa, Todd DeWees, Jeffrey D Bradley, Clifford G Robinson

**Affiliations:** 1 Department of Radiation Oncology, Mallinckrodt Institute of Radiology, Washington University in St. Louis, 4921 Parkview Place, Campus Box 8224, St. Louis, MO, 63110, USA; 2 Department of Radiology, Mallinckrodt Institute of Radiology, Washington University in St. Louis, St. Louis, MO, USA

**Keywords:** SBRT, Bronchioloalveolar carcinoma, Adenocarcinoma *in situ*, Minimally invasive adenocarcinoma, BAC, SABR

## Abstract

**Introduction:**

Ongoing prospective trials exploring stereotactic body radiation therapy (SBRT) for early stage non-small cell lung cancer (NSCLC) often exclude minimally invasive adenocarcinoma or adenocarcnioma *in situ*, formerly bronchioloalveolar carcinoma (BAC), due to concerns for accurate target delineation on CT. We performed a patterns of failure analysis to compare outcomes between BAC and other NSCLC subtypes.

**Methods:**

One hundred twenty patients with early stage NSCLC were treated with SBRT from 2004–2009. Pathologic confirmation of NSCLC was obtained in 97 patients. Radiotherapy was delivered according to RTOG guidelines. The log-rank test was used to compare outcomes between BAC and other NSCLC.

**Results:**

Median follow-up was 29 months. The median SBRT dose was 5400 cGy. Thirteen patients had radiographically diagnosed BAC and five patients had biopsy confirmed BAC, of which two had both. The three-year local control was 100% for biopsy-proven or radiographically diagnosed BAC (n = 18) and 86% for all other NSCLC subtypes (n = 102) (p = 0.13). Likewise, no significant difference was detected between BAC and other NSCLC for 3-year regional failure (12% vs. 20%, p = 0.45), progression-free survival (57.6% vs. 53.5%, p = 0.84) or overall survival (35% vs. 47%, p = 0.66). There was a trend towards lower three-year rates of freedom from distant failure in patients with any diagnosis of BAC compared to those without (26% vs. 38%, p = 0.053).

**Conclusions:**

Compared to other NSCLC subtypes, BAC appears to have similar patterns of failure and survival after treatment with SBRT, however there may be an increased risk of distant metastases with BAC. RTOG guideline-based target delineation provides encouraging local control rates for patients with BAC.

## Introduction

Stereotactic Body Radiotherapy (SBRT) has become a standard of care treatment for medically inoperable patients with early stage non-small cell lung cancer (NSCLC) [1]. Multiple prospective clinical trials have established the safety and efficacy of SBRT for the treatment of early stage NSCLC [2-9]. Fakiris et al. reported three year local control and cause specific survival rates of 88% and 82% respectively, and grade 3–4 toxicity rates of 10% for peripheral lung tumors [2]. RTOG 0236 reported similar findings for patients with peripheral tumors with three-year local control and disease free survival rates of 91% and 48% respectively, and grade 3–4 toxicity rates of 17% [3].

Bronchioloalveolar carcinoma (BAC), recently renamed as adenocarcinoma *in situ* or minimally invasive adenocarcinoma [10], is a sub-type of NSCLC with unique imaging characteristics and natural history relative to other sub-types. While invasive subtypes of BAC follow typical routes of lymphatic spread to hilar and mediastinal lymph nodes and metastasize outside of the thorax, non-invasive BAC frequently demonstrates a lepidic pattern of spread along alveoli, disseminating widely throughout the lung parenchyma [11]. In the United States, estimates of the incidence of BAC have varied from as high as 24 percent in a single institution series to less than 5 percent in a large series based upon the Surveillance, Epidemiology and End Results (SEER) database [12,13]. Pathologic confirmation of BAC is frequently unavailable for patients undergoing SBRT due to their inability to tolerate a needle biopsy because of medical comorbidities. In one single institution study of SBRT for medically inoperable lung cancer patients 29% of patients are treated without histologic confirmation [6]. In patients that do undergo biopsy, pathologists may not diagnose BAC unless the entire specimen is available. The inability to obtain histologic confirmation of BAC on a large percentage of patients undergoing SBRT has led to a frequent reliance on radiographic diagnosis of BAC.

Computed Tomography (CT) of the chest is the standard imaging test for the diagnosis of BAC. The variation in the patterns of tumor growth for BAC can lead to a variety of radiologic findings. In 40% of patients diagnosed with BAC, the tumor presents on CT imaging as solitary pulmonary nodules or masses [14]. The CT appearance of these lesions is a continuous spectrum between well-defined nodules and ground glass opacities (GGO) [15]. The GGO component of the tumor correlates with the lepidic growth pattern of the malignancy [16]. Lesions with both solid and GGO components suggest a mixed subtype of adenocarcinoma such as BAC with focal invasion and adenocarcinoma with BAC features [17]. An increasing size of the solid component correlates with a higher risk of an invasive component [14]. Lesions with GGO components on CT present a target delineation issue when treating early stage BAC with SBRT. There is currently no published literature on the appropriate way to design clinical target volumes for patients with BAC.

The utility of FDG-PET scanning in the diagnosis of BAC has recently been explored by multiple institutions. The sensitivity and specificity of FDG-PET in the diagnosis of BAC varies greatly depending on the presence of an invasive carcinoma component in the BAC. FDG-PET has been shown to miss up to 67% of BAC tumors without invasive components [18]. However, in cases of adenocarcinoma with BAC features, the diagnostic performance of FDG-PET was similar to other NSCLC [18]. The typical standardized uptake values (SUVs) of BAC are less than more virulent forms of NSCLC. This is likely due to BAC’s longer doubling time [19,20]. The median SUV of BAC lesions has been reported to be 2.5 which is also the typical threshold value for differentiating between and benign and malignant lesions [17]. BAC lesions with high SUV are more likely to have an invasive component and have a worse prognosis [21]. However, the typically low SUV of BAC lesions limits the usefulness of FDG-PET scanning in the diagnosis of BAC by resulting in a high false-negative rate [22].

In light of a perceived difficulty in target delineation on CT due to a propensity for a lepidic pattern of growth, ongoing prospective trials exploring SBRT for early stage NSCLC (RTOG 0618, RTOG 0915) have excluded BAC [23,24]. As such, reported outcomes from these studies cannot be used to infer utility of SBRT for BAC. Likewise, no other specific comparison in outcomes or patterns of failure has been made between BAC and other NSCLC histologies treated with SBRT to date. Therefore, we performed a comprehensive patterns of failure analysis to compare outcomes between BAC and other NSCLC subtypes.

## Materials and methods

An Institutional Review Board (IRB) - approved registry of patients undergoing lung SBRT from 2004–2009 at the Mallinckrodt Institute of Radiology was used for selection of 120 patients who were (1) treated with definitive intent for early stage disease, and (2) without history of malignancy in the preceding 2 years. Pathologic confirmation of NSCLC was obtained in 97 patients via CT guided biopsy or bronchoscopy. The remaining 23 patients did not undergo a biopsy attempt due to high clinical concern for pneumothorax. All patients underwent a chest CT scan, and the metastatic workup also included an abdominal CT scan (n = 45) and/or a [18 F] fluorodeoxyglucose (FDG) positron emission tomography (PET) scan (n = 112) for all patients. The CT studies of the chest were reviewed by a fellowship trained board certified thoracic radiologist (AJB). Diagnosis of BAC was based on imaging characteristics typical of BAC, such as ground glass nodule, solid nodule with ground glass halo, consolidation with air bronchograms, and nodules with pseudocavitation [25]. These imaging characteristics would correspond to adenocarcinoma *in situ* (AIS) and minimally invasive adenocarcinoma (MIA) (although there is overlap of the imaging features) [26]. BAC (currently AIS) was radiographically diagnosed if the nodule was purely ground glass. BAC (currently MIA) was also radiographically diagnosed if there was a ground glass nodule with a solid component or a nodule was present with pseudocavitation. Nodules that were completely solid, spiculated, and nodules with a CT angiogram sign were not given a diagnosis of BAC based on CT imaging characteristics.

The simulation and treatment planning procedures at our institution have been previously described [7]. Briefly, all patients underwent four-dimensional CT (4D-CT) without intravenous contrast using a 16 slice CT (Somatom Sensation 16, Philips Medical, Cleveland, OH) to determine tumor motion for target delineation. Patients were immobilized using either the Elekta Stereotactic Body Frame (SBF) (Elekta, Crawley, England) (n = 83), the BodyFIX® system (Medical Intelligence, Munich, Germany) (n = 28), or an Alpha Cradle (Smithers Medical Products, North Canton, OH) (n = 7). Two morbidly obese patients were immobilized using the VacLoc bag component of the BodyFIX® system alone. For patients immobilized with the SBF, an initial limited-field 4DCT was used to determine the longitudinal extent of tumor excursion during quiet respiration, and additional immobilization using diaphragmatic compression was applied for 30 patients (33%) with tumor excursions greater than 5 mm in the craniocaudal direction.

**Figure 1:**
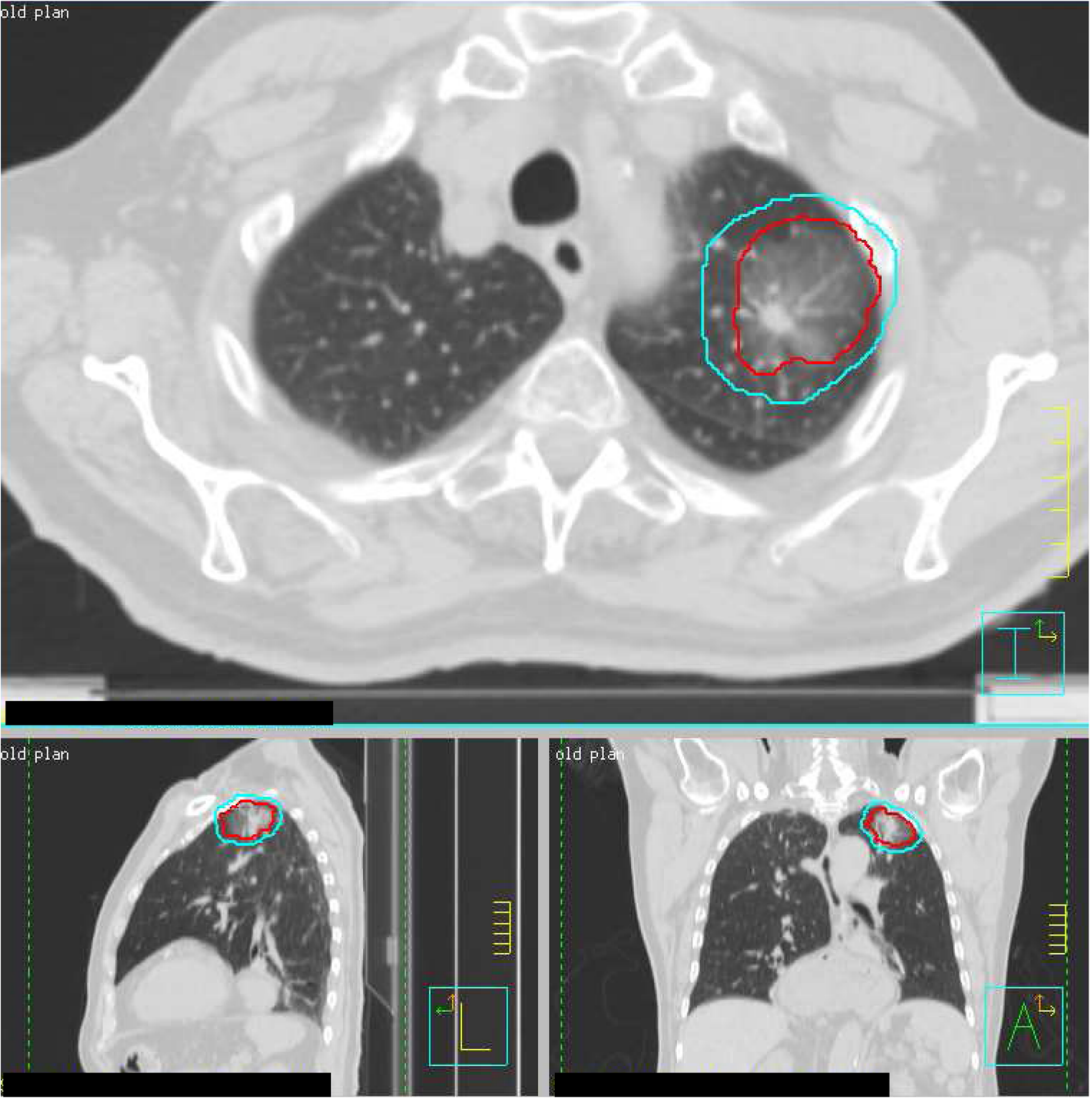
**Target volumes for a representative patient with BAC treated with SBRT.** Using a 4D CT simulation an ITV is created. A 0.5 cm margin is added to the ITV to create a PTV.

After co-registration of the 4D-CT to the simulation CT an internal target volume (ITV) was created to encompass the maximum intensity projection (MIP), and an additional PTV expansion was made following RTOG guidelines [27]. No additional measures were taken with regard to target delineation for suspected or proven BAC, nor were any modifications to the planning target volume (PTV) made other than those defined above (Figure 1). Prior to each treatment, tumors were localized using cone-beam CT image guidance and fluoroscopy was used confirm that the tumor was encompassed by the PTV when anatomically feasible.

Follow-up was performed with regular CTs of the chest without intravenous contrast according to institutional guidelines previously described [7]. Local control was defined as an absence of primary tumor progression (i.e., any response or stable disease) and absence of involved-lobe recurrence. FDG-PET/CT imaging was used in cases of suspected tumor relapse to aid differentiation between local recurrence and fibrosis. Nodal failures were defined as hilar or mediastinal nodal enlargement on CT with FDG-PET positivity, and distant failures were defined as any failure outside the thorax.

The survival and patterns of lung cancer failure in the patients with BAC was compared to all other patients with NSCLC treated with SBRT. The log-rank test was used to compare local control (LC), hilar failure, mediastinal failure, regional failure, freedom from distant failure (FFDF), progression free survival (PFS), and overall survival (OS), and was used for reported *p* values. Statistical analyses were performed using SAS® version 9.2.

## Results

One-hundred twenty patients that met study criteria were treated with SBRT between July 2004 and May 2009. Median follow-up was 29 months for all patients and 39 months for living patients. Median age was 74 years (range, 31–92), and 58 (48%) patients were female. Median SBRT dose was 5400 cGy in 3 fractions, and ranged from 4500 cGy to 6956 cGy in 3–6 fractions. Ninety-three patients (78%) were treated to 5400 cGy in 3 fractions and 11 patients (9%) received 5000 cGy in 5 fractions. The breakdown of NSCLC subtypes for 97 patients with pathologic confirmation of disease is shown in Table 1.

**Table 1:** Summary of radiologic and histologic diagnoses for patients with BAC and non-BAC NSCLC

**Histology**	**Biopsy**	**CT diagnosed**
**BAC**	**5**	**15**
**Non-BAC**	**91**	**105**
**Adenocarcinoma**	**33**	
**Squamous cell carcinoma**	**21**	
**NSCLC NOS**	**36**	
**Sarcomatoid**	**1**	
**Total**	**96**	**120**

Fifteen patients were deemed to have BAC based on pre-treatment chest CT. Of the 15 patients with radiological diagnosis of BAC, 3/15 did not have a biopsy, 6/15 had biopsy proven adenocarcinoma, 4/15 had biopsy proven NSCLC not otherwise specified, and 2/15 had biopsy proven BAC or adenocarcinoma with BAC features. Twelve of the fifteen patients with radiological diagnosis of BAC had GGOs. Two patients had a radiographical diagnosis of BAC but had a pathological diagnosis of squamous cell carcinoma, and thus were not included in the analysis. By combining patients with either a histologic (n = 5) or radiographic (n = 13) diagnosis of BAC a subgroup of eighteen patients with BAC was created for comparison with the entire study population.

**Figure 2:**
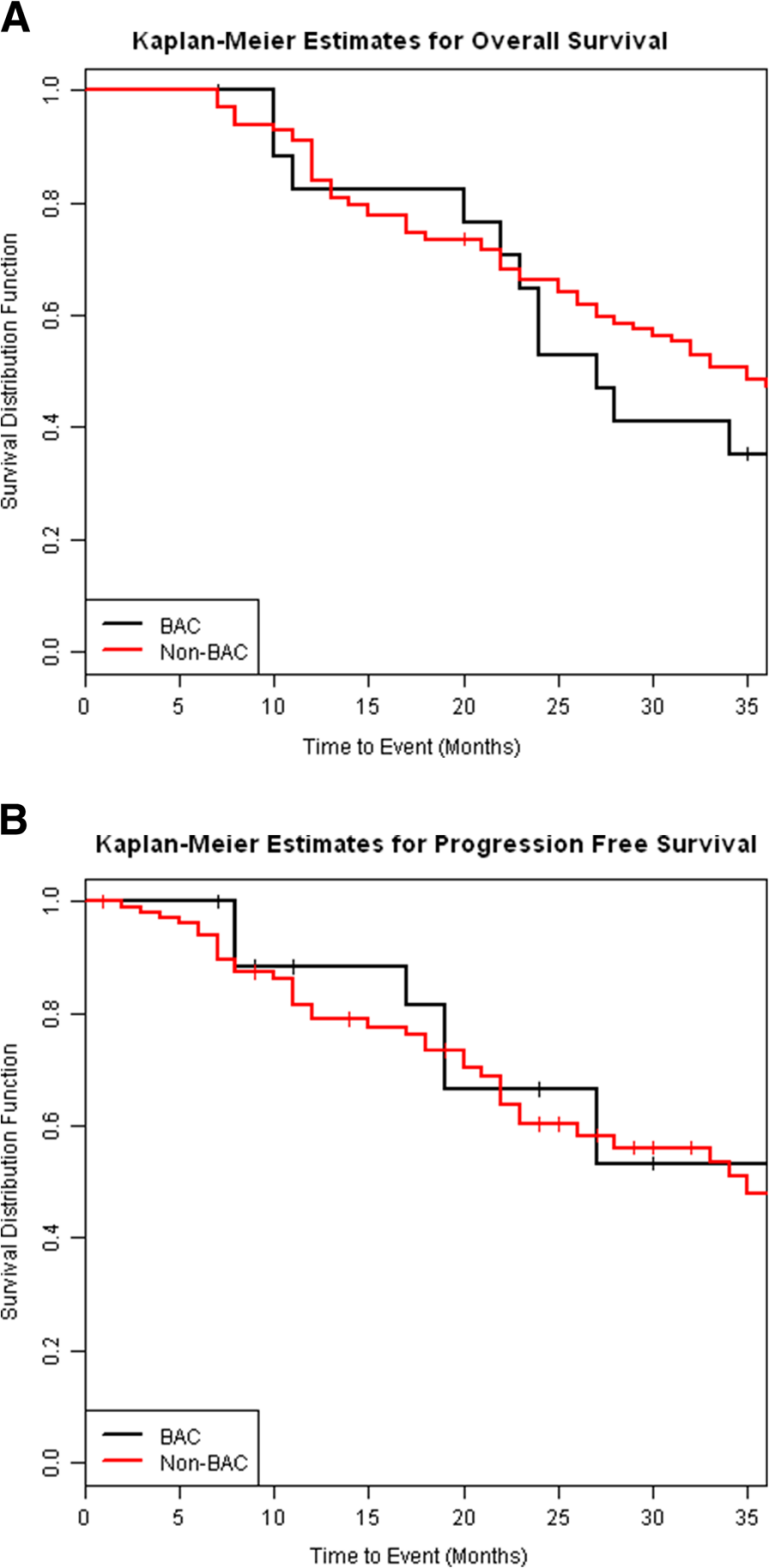
No significant difference was detected between BAC and non-BAC NSCLC for three-year (A) Overall Survival (35% vs 47%, p = 0.66) and (B) Progression Free Survival (53% vs 48%, p = 0.96).

**Figure 3:**
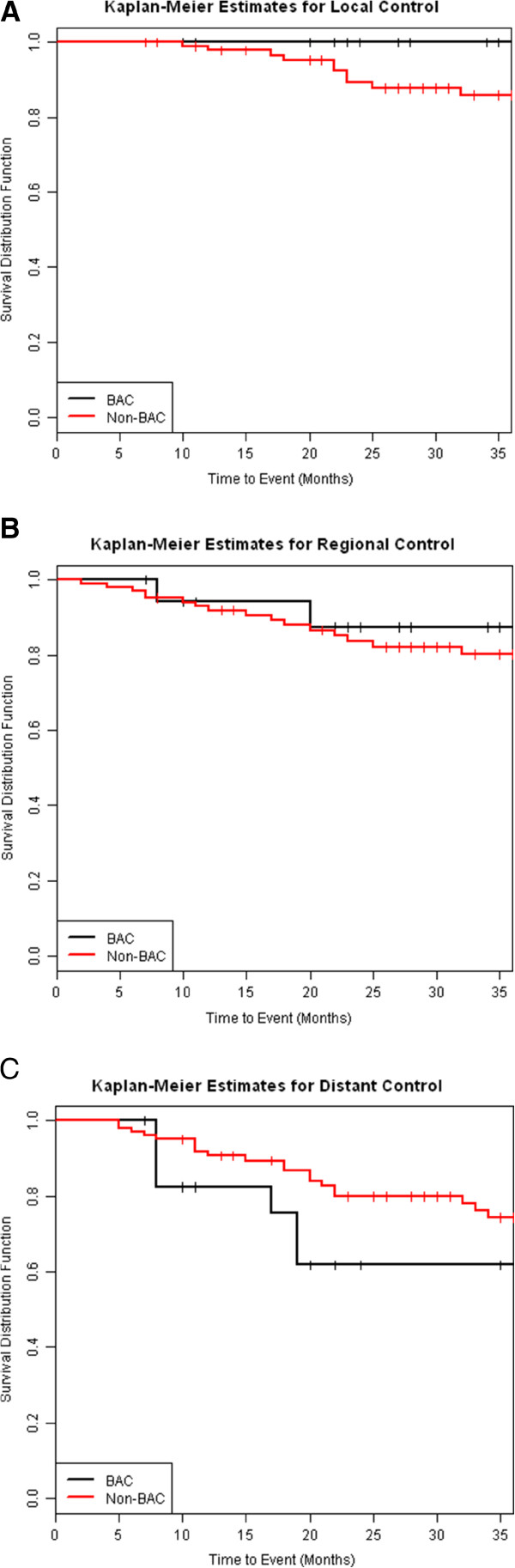
**No significant difference was detected between BAC and non-BAC NSCLC for three-year (A) Local Control (100% vs 86%, p = 0.13) (B) Regional Control (88% vs 80%, p = 0.45).** There is a trend toward lower three-year rates of distant control (26% vs 38%, p = 0.053) in patients with BAC vs non-BAC NSCLC **(C)**.

There was no difference in three-year overall survival (OS) (35% vs 47%, p = 0.66) or three-year progression-free survival (PFS) (53% vs 48%, p = 0.96) between patients with any (radiologic or histologic) diagnosis of BAC and those without (Figure 2). Similarly, no difference was seen in three-year rates of local control (defined as lack of tumor recurrence within the treated area) (100% vs 86%, p = 0.13), or regional (hilar or mediastinal) nodal control (88% vs 80%, p = 0.45) between patients with any diagnosis of BAC and those without (Figures 3A, 3B).

There was a trend toward lower three-year rates of distant control in patients with any diagnosis of BAC compared to those without (26% vs. 38%, p = 0.053) (Figure 3C). Nine of eighteen patients with any diagnosis of BAC developed metastases. Of those nine, four developed pulmonary metastases, four developed extrapulmonary metastases and one developed both. No significant difference was seen in three-year rates of pulmonary metastases (34% vs 17%, p = 0.17) or three-year rates of extrapulmonary metastases (21% vs. 18%, p = 0.23) between patients with any diagnosis of BAC and those without.

## Discussion

The patterns of failure of early stage NSCLC after SBRT have been reported by multiple institutions, however no institution to date has specifically reported the patterns of failure after SBRT for BAC [2,3,5,6,8,9]. Ongoing prospective trials exploring SBRT for early stage NSCLC (RTOG 0618, RTOG 0915) have excluded BAC [23,24]. Thus, reported outcomes from these studies cannot be used to infer utility of SBRT for BAC.

A large SEER analysis found that patients with bronchioloalveolar carcinoma that underwent lobectomy had improved overall survival compared to those that underwent wedge resection or segmentectomy [28]. However, prospective studies have found that wedge resection is potentially curative for patients with non-invasive BAC [29-31].

The pattern of failure after surgical removal of BAC was evaluated in a series of 93 patients with Stage I-IV histologically confirmed BAC that underwent wedge resection, lobectomy or pneumonectomy for their disease. Any lung recurrence was reported in 39 (42%) patients. Thirteen (33%) of the recurrences were multiple lung nodules, eight (21%) as a single nodule or mass, four as an infiltrating or pneumonic mass, four as miliary lesions, four as mixed alveolar and nodular opacity, four as lymphangitic spreading, and one each as a pleural effusion or rib destruction [32].

It is estimated that the risk of developing a second lung cancer in patients who survived resection of a non-small-cell lung cancer is approximately 1%-2% per patient per year [33]. Unless the patient’s second lung cancer is histologically unique it is difficult to ascertain whether the second lung cancer is a metastasis of the first cancer or a *de novo* cancer. This issue caused uncertainty in coding the patterns of failure after SBRT in our study. We elected to code multiple synchronous recurrences in the lung outside of the field of treatment as pulmonary metastases and a single new mass/nodule outside of the field of treatment as a second lung cancer.

We found that there is a trend towards an increased rate of metastases after SBRT in patient with a radiologic or histologic diagnosis of BAC compared to those with a diagnosis of non-BAC NSCLC (Figure 3C). This finding may be confounded by the known propensity for BAC to spread diffusely throughout the lungs. When considering only pulmonary metastases, there was no significant difference between patients with BAC compared to those with non-BAC NSCLC, but the results were limited by a smaller sample size.

As described above, patients with BAC were treated in an identical fashion as other patients with early stage NSCLC (Figure 1). We had no local failures amongst patients with BAC, and 12% rate of local failure (n = 12) in patients with non-BAC NSCLC which is consistent with the rates of local failure seen in SBRT series from other institutions [2-6,8,9]. There is a concern for marginal recurrence of BAC after less extensive local therapy such as a wedge resection instead of standard of care lobectomy. As mentioned previously a number of surgical series have found excellent local control rates after wedge resection for non-invasive BAC [29-31]. However, the local control rates after wedge resection for invasive Stage I NSCLC including those with a BAC component are not as favorable. A randomized trial of lobectomy versus a limited resection (wedge or segmentectomy) for T1 N0 NSCLC found increased rates of locoregional recurrence (17% vs 6%) and any recurrence (34% vs 26%) in patients who received a limited resection compared to those receiving lobectomy. These higher rates of recurrence led to a 50% increased rate of death with cancer and a 30% increase in overall death rate [34].

SBRT for localized BAC provides excellent local control and has similar patterns of failure vs. non-BAC NSCLC. Increased risk of distant metastases in this series is likely confounded by interpretation of new BAC versus true pulmonary metastases as implied by the similar rates of extrapulmonary failure. Patients with BAC should be considered for enrollment on ongoing and future prospective studies of SBRT.

## Competing interest

The authors declare that they have no competing interests.

## Authors’ contributions

SNB: Contributed to the creation of the database and drafted the final manuscript. AJB: Contributed to the creation of the database. JRO: Contributed to the creation of the database. KMC: Contributed to the creation of the database. AAG: Contributed to the creation of the database. TD: participated in the design of the study and performed the statistical analysis. JDB: conceived of the study, and participated in its design and coordination and helped to draft the manuscript. CGR: conceived of the study, and participated in its design and coordination and helped to draft the manuscript. All authors read and approved the final manuscript.
